# The use of PrP transgenic *Drosophila* to replace and reduce vertebrate hosts in the bioassay of mammalian prion infectivity

**DOI:** 10.12688/f1000research.14753.1

**Published:** 2018-05-15

**Authors:** Alana M. Thackray, Olivier Andréoletti, Raymond Bujdoso

**Affiliations:** 1 Department of Veterinary Medicine, University of Cambridge, Cambridge, CB3 OES, UK; 2 UMR INRA ENVT 1225 -Hôtes-Agents Pathogènes, Ecole Nationale Vétérinaire de Toulouse, Toulouse, 31076, France

**Keywords:** Prion, infectivity, bioassay, invertebrate, Drosophila

## Abstract

Prion diseases are fatal neurodegenerative conditions of humans and vertebrate species. The transmissible prion agent is a novel infectious particle composed principally of PrP ^Sc^, an abnormal isomer of the normal host protein PrP ^C^. The only reliable method to detect mammalian prion infectivity is by bioassay, invariably in a vertebrate host. The current prion bioassays typically involve intracerebral or peripheral inoculation of test material into the experimental host and subsequent euthanasia when clinical signs of terminal prion disease become evident. It may be months or years before the onset of clinical disease becomes evident and a pre-determined clinical end-point is reached. Consequently, bioassay of prion infectivity in vertebrate species is cumbersome, time consuming, expensive, and increasingly open to ethical debate because these animals are subjected to terminal neurodegenerative disease. Prions are a significant risk to public health through the potential for zoonotic transmission of animal prion diseases. Attention has focussed on the measurement of prion infectivity in different tissues and blood from prion-infected individuals in order to determine the distribution of infectious prions in diseased hosts. New animal models are required in order to replace or reduce, where possible, the dependency on the use of vertebrate species, including the ‘gold standard’ mouse prion bioassay, to assess prion infectivity levels. Here we highlight the development of a *Drosophila*-based prion bioassay, a highly sensitive and rapid invertebrate animal system that can efficiently detect mammalian prions. This novel invertebrate model system will be of considerable interest to biologists who perform prion bioassays as it will promote reduction and replacement in the number of sentient animals currently used for this purpose. This article is a composite of previous methods that provides an overview of the methodology of the model and discusses the experimental data to promote its viability for use instead of more sentient hosts.

Research highlights **Scientific benefits:**

- Displays higher levels of sensitivity than gold standard mouse bioassay

**3Rs benefits:**

- Can replace the use of rodent and other vertebrate species used in bioassays for mammalian prion infectivity

**Practical benefits:**

- Bioassay can be completed within approximately 6 weeks

**Current applications:**

- Assessment of mammalian prion infectivity

**Potential future applications:**

- Understanding the transmissibility of prion and prion-like proteins

## Introduction

Prion diseases, or transmissible spongiform encephalopathies (TSEs) are fatal, neurodegenerative conditions of humans and various vertebrate species ( Prusiner, 2004). These conditions include Creutzfeldt-Jakob disease (CJD) of humans, bovine spongiform encephalopathy (BSE) in cattle, scrapie in sheep, and Chronic Wasting Disease (CWD) of cervids. Prion diseases are a significant risk to public health because of their potential for zoonotic transmission, as evidenced by the BSE epizootic in UK cattle and subsequent appearance of variant CJD in humans ( Bruce *et al.*, 1997). The emergence of new prion diseases, such as atypical scrapie in sheep ( Benestad *et al.*, 2003) and atypical BSE in cattle ( Biacabe *et al.*, 2004; Casalone *et al.*, 2004), and new reservoirs of CWD in cervids ( Benestad *et al.*, 2016), pose fresh challenges to human food safety since their zoonotic potential is unknown. Consequently, much attention has been focussed on the detection of mammalian prion infectivity and its distribution in hosts affected by prion diseases. The goal of such studies is to understand the biology of infectious prions in order to alleviate their burden on animal health and to protect human health.

In contrast to conventional pathogens such as viruses and bacteria, prions lack a nucleic acid-based genome. Instead, the infectious prion agent comprises principally, if not solely, of PrP ^Sc^, a disease conformer of the normal host protein PrP ^C^ ( Prusiner, 1982). For these reasons prions are not detected by common molecular biology techniques, such as PCR. The only reliable method to measure prion infectivity is through bioassay in experimental hosts and various mammalian animal species have been used for this purpose. Prion infectivity studies in large experimental animals, such as primates, sheep and goats, have been instrumental in establishing core features of mammalian prion biology including disease transmissibility and the existence of different prion strains in a single PrP polypeptide ( Gajdusek *et al.*, 1966; Gajdusek *et al.*, 1968; Kimberlin, 1977; Kimberlin, 1982; Stamp, 1962). Cattle and cervids have been used for BSE and CWD pathogenesis in their natural hosts, respectively, in order to provide important information on the distribution of prion infectivity and the mechanisms of its spread in these ruminant species ( Mathiason *et al.*, 2006; Wells *et al.*, 1998).

Prion infectivity studies in large experimental hosts are hampered by long incubation times for the onset of clinical disease and the low numbers of animals used as a consequence of the difficulties in their housing, with resultant loss of statistical power. More robust and reproducible prion infectivity measurements were achieved with the discovery that sheep scrapie was experimentally transmissible to rodents ( Chandler, 1961; Zlotnik & Rennie, 1965), which allowed larger numbers of experimental animals to be used. Accordingly, mice, either wild type or those transgenic for PrP autologous to the species form of prions under study, hamsters, and bank vole have collectively been used for measurement of prion infectivity from many different TSE-affected hosts including humans ( Brandner & Jaunmuktane, 2017; Watts & Prusiner, 2014). However, even experimentally inoculated mice may take many months or years to develop prion disease and reach a pre-determined clinical end-point. Collectively, the bioassay of prion infectivity in vertebrate species is cumbersome, time consuming, financially very expensive, and increasingly open to ethical debate because these animals are subjected to terminal neurodegenerative disease.

In order to advance the principles of the 3Rs, namely replacement, reduction and refinement, with respect to animal experimentation in prion research, alternative methods to assess prion infectivity would be of significant benefit. Presently, *in vitro* cell culture systems do not exist that can detect natural isolates of important animal prion diseases such as BSE ( Oelschlegel *et al.*, 2015). Furthermore, while the *in vitro* amplification technique of protein misfolding cyclic amplification (PMCA) ( Saborio *et al.*, 2001) or QuIC ( Atarashi *et al.*, 2007) demonstrate the presence of abnormal PrP, they do not detect prion infectivity, which is only revealed by bioassay in an animal host. Since transmissibility is a defining hallmark of prion diseases, it is important to develop a reasonably rapid and versatile confirmatory prion infectivity bioassay to supplement *in vitro* biochemical-based prion diagnostic assays. The new prion bioassay is required to be as sensitive as the ‘gold standard’ mouse prion bioassay but preferably using a less sentient host and one that is less costly.

Here we present methodology and experimental data that describe the use of PrP transgenic *Drosophila* as a viable alternative to the employment of more sentient hosts to assess mammalian prion infectivity. Our methodology has allowed us to demonstrate that the *Drosophila*-based prion bioassay is extremely sensitive and can detect a ≥10 ^-10^-fold dilution of scrapie-infected sheep brain homogenate ( Thackray *et al.*, 2016), a significantly higher level of sensitivity compared to the ‘gold standard’ mouse prion bioassay ( Andreoletti *et al.*, 2011). Furthermore, our fly-based prion bioassay can be completed within ≈6 weeks, in contrast to vertebrate species that may require months or years to assess the same prion inocula. In addition, we have shown that PrP transgenic *Drosophila* can detect prion-infected blood from asymptomatic scrapie-infected sheep ( Thackray *et al.*, 2016). This suggests this novel invertebrate system has significant practical use as a potential confirmatory blood test for prion diseased individuals, for example humans with vCJD.

This article is a composite of previously published methods to highlight the development of PrP transgenic *Drosophila* for use as a new prion bioassay for the sensitive and rapid assessment of mammalian prion infectivity. In doing so, we stress the utility of *Drosophila* to model transmissible mammalian prion disease. This new animal model will be of considerable interest to experimentalists who perform prion bioassays as it will allow reduction and partial replacement, where possible, in the number of sentient hosts currently used for this purpose.

## Methods

### Development of a *Drosophila*-based bioassay for mammalian prion infectivity

***Overview.*** We have generated *Drosophila* transgenic for topological variants of mature length ovine PrP by pUAST / PhiC31-mediated site-directed mutagenesis. These fly lines, generated by Bestgene (California, USA), were transgenic for ovine PrP together with an N-terminal leader peptide and a C-terminal GPI signal sequence [PrP(GPI)] ( Thackray *et al.*, 2012c; Thackray *et al.*, 2014a), or expressed ovine PrP without either an N-terminal leader peptide or a C-terminal GPI signal sequence [PrP(cyt)] ( Thackray *et al.*, 2014b). PCR and DNA sequencing was used in order to confirm that each PrP transgene was present as a single copy and located at the single 51D-site in the fly genome. We subsequently removed the red fluorescent protein (RFP) cassette located at the 51D site of the fly genome by *Cre-*mediated cleavage in each PrP fly line. Confocal microscopy of *Drosophila* S2 cells transiently transfected with the different pUAST-VRQ variants showed that PrP(GPI) and PrP(ΔGPI) entered the secretory pathway, whereas PrP(cyt) was restricted to the cytosol ( Thackray *et al.*, 2014a). Non-RFP *UAS*-PrP transgenic fly lines were subsequently crossed with *GAL4*-driver lines to allow expression of PrP in *Drosophila*.

PrP transgenic *Drosophila* were exposed to mammalian prions at the larval stage ( Thackray *et al.*, 2012b; Thackray *et al.*, 2014a; Thackray *et al.*, 2014b; Thackray *et al.*, 2016). After hatching, *Drosophila* were transferred to prion-free culture tubes. At various time points during their adult lifespan, groups of *Drosophila* were analysed for locomotor ability, or euthanised, decapitated and homogenate prepared from the isolated fly heads ( Thackray *et al.*, 2012b; Thackray *et al.*, 2014a; Thackray *et al.*, 2014b; Thackray *et al.*, 2016). These homogenates were used to seed *in vitro* PMCA reactions in order to reveal the presence of prion seeding activity ( Thackray *et al.*, 2014a); SDS/PAGE western blot detection of PrP ^Sc^; RNASeq-based transcriptome analysis ( Bujdoso *et al.*, 2015); or used in fly-to-fly or fly-to-mouse prion transmission studies ( Thackray *et al.*, 2016).

***Fly stocks.*** The following fly lines were obtained from the Department of Genetics, University of Cambridge, UK.

- *Actin-5C-GAL4* (y w; P{w[+mC]=Act5C-Gal4}25F01/CyO, y[+])
- *Elav-GAL4* (P{w[+mW.hs]=GawB}elav[C155])
- 51D (w; M{3xP3-RFP.attP}ZH-51D)

*Cre*-mediated removal of the RFP gene from the VRQ and 51D fly genome was performed by conventional fly crosses ( Thackray *et al.*, 2014a). PrP transgenic *Drosophila* were crossed with either the *Elav-GAL4* or *Actin-5C-GAL4* driver fly lines to derive transgenic flies that expressed PrP pan neuronally or ubiquitously, respectively. 51D *Drosophila* crossed with either driver fly line were used as control flies where appropriate. All fly lines were raised on standard cornmeal media at 25°C and maintained at low to medium density, and pre-mated before experimental use.

### Prion inoculation of *Drosophila*

***Primary transmission of sheep scrapie (sheep-to-fly).*** *Drosophila* at the larval stage of development were exposed to brain homogenate of cerebral cortex tissue from a confirmed VRQ/VRQ PG127 (alternatively referred to as DAW or G _338_) scrapie-positive sheep (SE1848/0005) ( Thackray *et al.*, 2008) or blood plasma from scrapie-positive sheep ( Lacroux *et al.*, 2012; Thackray *et al.*, 2016). New Zealand-derived VRQ/VRQ scrapie-free brain tissue or blood plasma were used as control material. Two hundred and fifty microlitres of either 10% (v/v) blood plasma or 1% (w/v) of sheep brain homogenate, or a 1/10 dilution series (v/v) of these samples, prepared in PBS pH7.4, were added to the top of the cornmeal that contained third instar *Drosophila* larvae in 3-inch plastic vials. Following eclosion (i.e. hatching) flies were transferred to fresh non-treated vials.

***Secondary transmission of sheep scrapie (fly-to-fly).*** *Drosophila* head homogenates were prepared from 30 day old flies that had been exposed at the larval stage to scrapie-positive or scrapie-negative sheep brain material. Two hundred and fifty microlitres of a 10 ^-1^ (v/v) dilution of the original fly brain homogenate were added to the top of the cornmeal that contained third instar *Drosophila* larvae in 3-inch plastic vials. In all cases, flies were transferred to fresh, non-treated vials following eclosion.

### *Drosophila* model characterisation and validation

***Preparation of Drosophila head homogenate.*** Whole flies in an eppendorf tube were frozen in liquid nitrogen for 10 minutes and then vortexed for 2 minutes to cause decapitation. Individual fly heads were isolated and placed in clean eppendorf tubes using a fine paint brush. PBS pH 7.4 was added to give 1µL / head and homogenates were prepared by manual grinding of the fly heads with sterilised plastic pestles. For western blot analysis, fly head homogenate was mixed with an equal volume of 20% scrapie-free sheep brain homogenate prior to extraction and PK digestion as previously described ( Lacroux *et al.*, 2012) using monoclonal antibody Sha31 ( Feraudet *et al.*, 2005).

***Protein misfolding cyclic amplification (PMCA).*** PMCA was carried out as previously described ( Lacroux *et al.*, 2012). The substrate consisted of 10% (w/v) ovine VRQ PrP (tg338) transgenic mouse brain homogenate in PBS pH 7.4, 0.1% Triton X-100 and 150 mM NaCl buffer ( Lacroux *et al.*, 2014). Five µL of fly head homogenate were mixed with 45µL of substrate in 0.2 mL thin wall PCR tubes. Sealed tubes were then placed in the horn of a Misonix 4000 sonicator for one round of 96 cycles. Each cycle consisted of a 10 second sonication step (70% of power) followed by a 14 minute and 50 second incubation step. Twenty µL of each reaction mix were subsequently treated with PK (4µ g of PK per mg of protein) at 37°C for 2 hours and the reaction stopped by adding Pefabloc (4mM final concentration). PK-resistant PrP was detected by western blot as previously described ( Lacroux *et al.*, 2012) using monoclonal antibody Sha31 ( Feraudet *et al.*, 2005).

***Fly-to-mouse prion transmission.*** Fly-to-mouse prion transmission was carried out in ovine VRQ PrP (tg338) transgenic mice ( Le Dur *et al.*, 2005), which are highly efficient for the detection of ovine prion infectivity. All mouse bioassays were performed under licence number D-31-555-27, in compliance with institutional and national guidelines including ethical approval, and in accordance with the protection of animals used for scientific purposes under European Community Council Directive 2010/63/UE. Female tg338 mice (n=6) bred in-house aged 12 – 14 weeks were housed in a single cage with environmental enrichment and maintained under controlled conditions with respect to lighting, temperature, humidity and noise. Mice were injected intracerebrally with 20µL of diluted fly head homogenate (to give approximately 2 fly head equivalents per mouse) and monitored daily until the occurrence of clinical signs of mouse prion disease. Inoculated mice were euthanised when they started to show locomotor disorders and any impairment in their capacity to feed, or at a pre-defined end-point for the assay (>250 days) ( Andreoletti *et al.*, 2011). Brain tissue (cerebral cortex) was collected from euthanised mice and frozen for PrP ^Sc^ analysis by Western blot (TeSeE, BioRad) or PET blot analysis ( Andreoletti *et al.*, 2011).

### Negative geotaxis climbing assay

The locomotor ability of flies was assessed in a negative geotaxis climbing assay initiated with 45 (3 × n=15) age-matched, pre-mated female flies in each treatment group ( Nichols *et al.*, 2012; Thackray *et al.*, 2014a; Thackray *et al.*, 2014b). *Drosophila* were placed in adapted plastic 25mL pipettes that were used as vertical climbing columns and allowed to acclimatise for 30 minutes prior to assessment of their locomotor ability. Flies were tapped to the bottom of the pipette (using the same number and intensity of taps on each occasion) and then allowed to climb for 45 seconds. At the end of the climbing period the number of flies above the 25mL mark, the number below the 2mL mark and the number in between the 2mL and 25mL mark was recorded. This procedure was performed three times at each time point. The performance index (PI) was calculated for each group of 15 flies (average of 3 trials) using the formula: PI = 0.5 × ( *n*total + *n*top – *n*bottom)/ *n*total where *n*total is the total number of flies, *n*top is the total number of flies at the top, and *n*bottom is the total number of flies at the bottom. A PI value of 1 is recorded if all flies climb to the top of the tube whereas the value is 0 if no flies climb the tube past the 2mL mark. The mean PI ± SD at individual time points for each treatment group was plotted as a regression line.

Detailed methodology of the climbing assay is as follows:

*Preparation of climbing assay pipettes*

Plastic 25mL pipettes used in the climbing assay were prepared by taking a sharp saw blade and carefully cutting the top off the pipette. The cut edges were filed down in order to prevent damage to the *Drosophila* wings when the flies were added to, or removed from, the pipettes before or after the climbing assay was carried out. The tip of each pipette was sealed with a small piece of nescofilm wrapped securely around the point in order to prevent the escape of *Drosophila* during the assay. Clean cotton wool plugs were pushed into the top of each pipette to ensure a close fit so that the *Drosophila* could not climb out of the pipette once the assay had started.

*Addition of flies to the climbing assay pipettes*

At the start of each assay, the flies were counted in each set of fly vials dedicated to each treatment group in order to verify the number present (typically 3 vials, each containing 15 flies at the start of the experiment). The *Drosophila* from one vial were gently tipped into the top of a pipette using a dedicated plastic funnel for each treatment group. The cotton wool plug was securely fitted to stopper the top of the pipette as soon as the funnel was removed. The *Drosophila* were tapped to the bottom of the pipette, which was then laid horizontal and the flies allowed to acclimatise prior to the assay.

*Acclimatisation of flies in the climbing assay pipettes*

The pipettes that contained the *Drosophila* were placed horizontal at 25°C for 30 minutes in order to allow the flies to acclimatise. After the acclimatisation period the *Drosophila* were ready to start the climbing assay.

*Pre-test climbing assay procedure*

The tip of the climbing assay pipette was gently tapped a sufficient number of times on the bench to gather the flies together at the bottom of the apparatus, which was subsequently placed in a tube rack in an upright position at room temperature. The flies were allowed to climb for 45 seconds. There was no recording of data from this run as its purpose was to allow the flies to ‘practice’ climbing in the pipette that was held in an upright position.

*Actual climbing assay procedure*

Once the pre-test procedure had been completed, the climbing assay pipette was tapped gently on the bench (using the same number and intensity of taps as for the pre-test) and the apparatus was placed upright in the tube rack at room temperature. *Drosophila* were allowed to climb for 45 seconds. During the 45 seconds the number of flies to climb above the 2mL and 25mL marks were recorded. At the end of the 45 seconds, the number of flies above the 25mL mark, the number below the 2mL mark and the number in between the 2mL and 25mL mark was recorded. The whole climbing assay was repeated 2 more times to give a total of 3 readings per pipette.

*End of climbing assay procedure*

When all 3 climbing assay procedures had been performed, the *Drosophila* were gently tapped away from the cotton wool plug so it could be removed from the pipette without the loss of any flies. The *Drosophila* were then returned to fresh food vials using the dedicated plastic funnel for each group. Once the flies were back in fresh culture vials, the numbers of flies were counted and the number recorded on the lid to confirm that no flies had been lost during the assay or during the transfer to or from the pipettes. Climbing assay pipettes were checked to ensure no flies were stuck in the bottom of the pipette. The culture vials were returned to 25°C for routine fly maintenance.

## Results and Discussion

### PrP transgenic *Drosophila* to bioassay mammalian prion infectivity

*Drosophila* have proven to be a versatile experimental invertebrate host for use in the study of mammalian neurodegenerative diseases ( Bilen & Bonini, 2005; Lu & Vogel, 2009). Several important features of *Drosophila* have aided this development. Firstly, *Drosophila* and mammals show conservation of basic components of the nervous system ( Hirth & Reichert, 1999); Secondly, the genetics of *Drosophila* are well-defined, which allows the generation of transgenic flies with tissue-specific transgene expression. Third, the normal physiology and development of *Drosophila* is sufficiently well established to allow the use of behavioural assays that detect neurotoxicity in the living organism ( Marsh & Thompson, 2006). Fourth, large numbers of *Drosophila* are readily generated in a short time and since this organism has a relatively short life span allows the rapid collection of, statistically robust data ( Piper *et al.*, 2005).

In order to develop an invertebrate-based bioassay for mammalian prion infectivity, we have generated ovine PrP transgenic *Drosophila* and have assessed the ability of these flies to detect ovine scrapie prions.

### Generation of PrP transgenic *Drosophila* and prion inoculation

The *Drosophila* genome does not contain an orthologue of mammalian PrP and cellular expression of this protein is required for prion-induced neurotoxicity, which occurs during prion replication ( Büeler *et al.*, 1993; Mallucci *et al.*, 2003). We exploited the successful application of PrP transgenesis to modify the susceptibility of a host for prion replication ( Crozet *et al.*, 2001; Thackray *et al.*, 2012a; Vilotte *et al.*, 2001) in order to explore *Drosophila* as a new animal model to assess mammalian prion infectivity.

Although PrP ^C^ is primarily attached by a GPI anchor to the external side of the cell membrane, topological variants of the protein, including cytoplasmic and secreted forms, can arise during its biogenesis and metabolism ( Borchelt *et al.*, 1993; Chakrabarti *et al.*, 2009; Hay *et al.*, 1987; Hegde *et al.*, 1998; Kim & Hegde, 2002; Stewart & Harris, 2003; Taylor *et al.*, 2009). The role of these different forms of PrP in prion-mediated toxicity is not fully clarified. Accordingly, we generated *Drosophila* transgenic for the mature form of ovine PrP (amino acid residues 25 – 232) that was flanked by an N-terminal leader peptide and a C-terminal GPI signal peptide, which allowed expression of ovine PrP in the fly that was targeted to the plasma membrane, hereafter referred to as PrP(GPI) ( Thackray *et al.*, 2012c; Thackray *et al.*, 2014a). In addition, we generated *Drosophila* transgenic for the mature form of ovine PrP that lacked the N-terminal leader peptide and C-terminal GPI signal peptide, which restricted PrP expression to the cytoplasm, hereafter referred to as PrP(cyt) ( Thackray *et al.*, 2014b). In order to generate *Drosophila* transgenic for these PrP variants we employed pUAST / PhiC31-mediated site-directed mutagenesis, whereby a single copy of the transgene of interest is delivered to the same landing-site in the fly genome in each respective fly line. Using this strategy, we demonstrated that different genotypes of ovine PrP protein could be successfully expressed in *Drosophila*. Expression of these ovine PrP variants in *Drosophila* had no adverse phenotypic effect upon the fly.

We subsequently tested the hypothesis that PrP transgenic *Drosophila* could bioassay exogenous ovine prions. To do so, *Drosophila*, at the larval stage, were exposed to sheep scrapie material known to contain prion infectivity as determined previously by transmission studies in mice ( Thackray *et al.*, 2008). Control inoculum consisted of known scrapie-free sheep brain homogenate. *Drosophila* were inoculated with scrapie-infected or scrapie-free sheep brain homogenate by addition of the material to larval feed. After hatching, flies were transferred to prion-free tubes and maintained for ≥40 days, during which time they were analysed for hallmark features of mammalian prion disease, namely the accumulation of infectious prions and evidence of a toxic phenotype.

### Accumulation of prions in scrapie-exposed PrP transgenic *Drosophila*

We first investigated whether scrapie-exposed PrP transgenic *Drosophila* accumulated prions by measurement of prion seeding activity, a surrogate marker of PrP ^Sc^, using *in vitro* PMCA. Head homogenate prepared from scrapie-exposed, and control flies, was used as seed in PMCA together with brain homogenate from ovine PrP transgenic (tg338) mice as substrate. After amplification, the reaction mix was subjected to Proteinase K digest (PK) and the products analysed by western blot using an anti-PrP monoclonal antibody. Significantly, only reaction products of tubes seeded with head homogenate from scrapie-exposed PrP transgenic *Drosophila* showed the presence of PK-resistant PrP ^Sc^, which was good evidence for the presence of disease-associated PrP in the brains of these flies ( Thackray *et al.*, 2014a). This was supported by the presence of a potentially misfolded conformer of PrP evident by immunohistochemistry in scrapie-exposed ovine PrP transgenic *Drosophila* and insoluble PrP accumulation in these flies detected by conformation dependent immunoassay ( Thackray *et al.*, 2012b).

We next investigated whether *bona fide* infectious prions accumulated in scrapie-exposed *Drosophila*. This was addressed by fly-to-mouse transmission studies using ovine PrP transgenic (tg338) mice ( Thackray *et al.*, 2016). Remarkably, tg338 mice inoculated with head homogenate from scrapie-exposed PrP transgenic *Drosophila* developed mouse prion disease with 100% attack rate with a relatively rapid incubation time, indicative of a reasonably high level of prion infectivity in the fly head homogenate. The lack of detectable prion infectivity in head homogenate from scrapie-exposed control non-transgenic *Drosophila* argued against persistence of inoculum being responsible for the observed fly-to-mouse prion transmission ( Thackray *et al.*, 2016).

### Prion-induced toxicity in scrapie-exposed PrP transgenic *Drosophila*

The presence of prion infectivity in scrapie-exposed PrP transgenic *Drosophila* was indicative of prion replication in these flies. Since prion-induced neurotoxicity occurs concomitantly with prion replication in mammalian hosts ( Büeler *et al.*, 1993; Mallucci *et al.*, 2003), we investigated whether scrapie-exposed PrP transgenic *Drosophila* demonstrated a toxic phenotype. We assessed whether prion-exposed *Drosophila* showed any movement defects, since clinical signs of scrapie infection in sheep include locomotor defects, such as ataxia ( Jeffrey & Gonzalez, 2007). To do so, we performed a negative geotaxis climbing assay ( Nichols *et al.*, 2012; Thackray *et al.*, 2014a; Thackray *et al.*, 2014b) using adult *Drosophila* exposed at the larval stage to ovine scrapie. After hatching, scrapie-exposed ovine PrP transgenic *Drosophila* showed an accelerated decline in locomotor activity. The severity of the locomotor defect increased as the flies aged, indicative of progressive illness. We also assessed whether scrapie-exposure affected the survival of PrP transgenic *Drosophila* since mammalian prion diseases are invariably fatal in affected individuals. Following exposure to scrapie material at the larval stage, adult PrP transgenic *Drosophila* showed a significantly enhanced mortality rate ( Thackray *et al.*, 2012b).

Collectively, these findings demonstrated that scrapie-exposed ovine PrP transgenic *Drosophila* accumulated prions that were transmissible to a mammalian host. Prion accumulation in the fly was associated with a progressive toxic phenotype evident as a locomotor defect. These hallmark features of mammalian prion disease in the fly were prion-mediated and PrP dependent since the effects were not observed in PrP transgenic *Drosophila* exposed to normal sheep brain material and were not displayed by scrapie-exposed flies that lacked PrP expression. These observations show that PrP transgenic *Drosophila* can be used to bioassay mammalian prion infectivity.

### Sensitivity of PrP transgenic *Drosophila* to exogenous prions

In order to determine the sensitivity of the fly-based prion bioassay, ovine PrP transgenic *Drosophila*, at the larval stage, were exposed to a 1/10 (v/v) dilution series of scrapie-infected sheep brain homogenate. After hatching, the locomotor ability of adult prion-exposed *Drosophila* was assessed by a negative geotaxis climbing assay as the flies aged. We observed that the accelerated decline in locomotor ability displayed by adult PrP transgenic *Drosophila* diminished upon exposure to increasing dilution of scrapie-infected brain homogenate at the larval stage ( Thackray *et al.*, 2016). A statistically significant decline in locomotor ability was induced in PrP transgenic *Drosophila* by dilutions of scrapie-infected sheep brain homogenate in the range 10 ^-2^ - 10 ^-10^. For comparative purposes, we have used ovine PrP transgenic (tg338) mice to bioassay sheep scrapie-infected brain material. The tg338 mouse prion bioassay was able to detect sheep scrapie inoculum diluted to 10 ^-5^, with the most dilute sample detected after a time course of ≈120 days in this mouse line ( Andreoletti *et al.*, 2011). These data showed that the *Drosophila*-based prion bioassay is of the order 10 ^5^-fold more sensitive than the tg338 mouse prion bioassay and can be completed in a significantly shorter time frame.

### Detection of prion-infected blood by PrP transgenic *Drosophila*

The high level of sensitivity shown by ovine PrP transgenic *Drosophila* for ovine prions suggested the fly bioassay would be able to detect the low level of prion infectivity present in the blood of prion-diseased individuals. We tested this hypothesis by inoculating ovine PrP transgenic *Drosophila* with plasma samples from sheep experimentally infected with scrapie ( Thackray *et al.*, 2012b). We decided to bioassay plasma since this particular blood fraction has been reported to contain low levels of prion infectivity and has proven to be difficult to assess by conventional prion bioassay ( Lacroux *et al.*, 2012; Mathiason *et al.*, 2010). We found that PrP transgenic *Drosophila* developed an accelerated decline in locomotor activity that became progressively reduced after exposure to more dilute samples of scrapie-infected plasma ( Thackray *et al.*, 2016). These observations were suggestive of titration of a particulate transmissible moiety in plasma obtained from scrapie infected sheep, a distinctive feature of the infectious scrapie agent ( Stamp, 1962). The sheep plasma samples were known to contain scrapie prion infectivity as they had previously been transmitted to sheep and mice ( Lacroux *et al.*, 2012).

We also observed that plasma isolated from natural scrapie-infected sheep could induce a toxic phenotype in ovine PrP transgenic flies ( Thackray *et al.*, 2016). The response to natural scrapie plasma was evident with samples collected from asymptomatic scrapie-infected sheep aged ≥6 months of age and was more pronounced after exposure to plasma obtained during the clinical phase, which commenced around 20 months of age. Importantly, we determined through fly-to-fly transmission that the toxic fly phenotype induced by pre-clinical natural scrapie plasma was transmissible ( Thackray *et al.*, 2016). These observations showed that ovine PrP transgenic *Drosophila* could successfully bioassay a transmissible moiety in the blood of scrapie-infected sheep, which was detectable at an early pre-clinical time point.

Transfusion experiments in sheep show that whole blood from non-clinical ovine donors aged ≥3 months can be used to detect scrapie-infected animals ( Lacroux *et al.*, 2012). We consider that PrP transgenic *Drosophila* show a similar, if not greater, sensitivity than transfusion studies in the natural host since plasma from scrapie-affected sheep contains less prion infectivity than whole blood ( Lacroux *et al.*, 2012). Furthermore, the amount of time required to bioassay plasma in PrP transgenic *Drosophila* was significantly shorter than the case for transfusion studies in the natural host ( McCutcheon *et al.*, 2011).

## Conclusion

Many advances in prion biology have been inextricably linked to the use of experimental animals; either to model prion diseases in general or to assess prion infectivity *per se*. We have demonstrated that core features of mammalian prion disease, namely accumulation of disease-associated PrP and development of a transmissible toxic phenotype, can be re-capitulated in prion-exposed PrP transgenic *Drosophila*. Significantly, we have shown that ovine PrP transgenic *Drosophila* proved to be more sensitive, by several orders of magnitude, and more rapid than the ‘gold standard’ mouse bioassay for the detection of sheep scrapie prions.

These observations support the use of PrP transgenic *Drosophila* as a new animal system to contribute to the study of mammalian prion disease. For example, the ease of transgenesis in *Drosophila* will allow the development of fly lines that express different species forms of PrP, such as human, bovine and cervid PrP, in order to address important questions on the pathogenic potential of other possible zoonotic prions, such as those associated with atypical BSE and CWD. *Drosophila* are already used to model other protein misfolding neurodegenerative diseases. This provides considerable expertise within the scientific community to assist with the development of this tractable experimental host in an important area of animal and human health. As such, there are no significant impediments to the use of PrP transgenic *Drosophila* in mammalian prion disease studies.

Accordingly, suitable uptake of the fly prion bioassay will be expected to have a considerable impact on the reduction and replacement, where appropriate, of more sentient hosts in the assessment of mammalian prion infectivity. In addition, the use of a *Drosophila*-based prion bioassay will provide a considerable refinement of the experimental protocols used to assess prion diseases. In this context, the use of *Drosophila* to assess mammalian prion infectivity would appear to have considerable advantages over more sentient species currently used for this purpose. Furthermore, translatability of this new invertebrate model of mammalian prion disease will be expected to provide a proof-of-concept to aid the development of new animal systems to study the prion-like properties of other neurodegenerative disease-related proteins, such as amyloid beta and tau.

## Data availability

All data underlying the results presented throughout this article are available from previous publications, which have been referenced appropriately.
