# Supplementary material for: The use of PrP transgenic Drosophila to replace and reduce vertebrate hosts in the bioassay of mammalian prion infectivity
Source: F1000Res. 2018 May 15;7:595. [Version 1] doi: 10.12688/f1000research.14753.1 (PMC5998032; doi:10.12688/f1000research.14753.1)
Supplement: Peer review history

# Peer review: Reviewer response for version 1

**DOI:** 10.5256/f1000research.16051.r34029

**Published:** 2018-06-12

**Authors:** Siddharthan Chandran, James Alibhai

**Affiliations:** 1 The University of Edinburgh, Edinburgh, UK; 2 Centre for Clinical Brain Sciences, The University of Edinburgh, Edinburgh, UK

This paper by Thackray *et al.* provides an interesting and useful overview of the development and advantages of utilising alternate methods of investigating prion infectivity, such as the Drosophila protocols outlined in this paper. The ethical implications of the paper are well discussed as are the scientific advantages of the method over the ‘gold standard’ murine studies. Overall this paper is well written, concise and a good review of the previous literature of prion-infected Drosophila research. Specific comments:

1. Page 3, paragraph 4 – The authors should consider the fact that cell culture methods have been used to replicate and define human prions in human cell which have also shown capability of sub-passage between cultures ^1^. Also recommend reference to this paper later alongside animal ‘gold standard’ comments as the sensitivity of the Drosophila model is unrivalled by human cell culture also and thus supports the Drosophila method.
2. Page 4, preparation of drosophila head homogenate – be more explicit about how fly heads were isolated after vortex. Assume this is done manually? Are there technical issues with other parts of the fly torso impeding recognition of the fly head? If so, how do you overcome this (or does it matter)?
3. Page 5, acclimatisation of flies in the climbing assay pipettes – this section is repetitive to the sentence above. Recommend deleting and moving information about the 30 minute incubation at 25C to sentence above.
4. Recommend addition of simple figures that visualise the sheep à fly and fly à fly experiments with the results (or a cartoon depiction of the results).

**Are a suitable application and appropriate end-users identified?**

 Yes

**If applicable, is the statistical analysis and its interpretation appropriate?**

 Not applicable

**Are the 3Rs implications of the work described accurately?**

 Yes

Is the rationale for developing the new method (or application) clearly explained?

Yes

Is the description of the method technically sound?

Yes

Are the conclusions about the method and its performance adequately supported by the findings presented in the article?

Yes

If any results are presented, are all the source data underlying the results available to ensure full reproducibility?

Yes

Are sufficient details provided to allow replication of the method development and its use by others?

Yes

Reviewer Expertise:

Referee suggested by the NC3Rs for their scientific expertise and experience in assessing 3Rs impact. Additional expertise: Prion disease. Rodent – Human Models.

We confirm that we have read this submission and believe that we have an appropriate level of expertise to confirm that it is of an acceptable scientific standard.

## References

[1] : Human stem cell-derived astrocytes replicate human prions in a PRNP genotype-dependent manner. *J Exp Med*.2017;214(12) : 10.1084/jem.20161547 3481-3495 10.1084/jem.20161547 29141869PMC5716027

# Peer review: Reviewer response for version 1

**DOI:** 10.5256/f1000research.16051.r34033

**Published:** 2018-05-29

**Authors:** Janne M Toivonen

**Affiliations:** 1 LAGENBIO, Veterinary Faculty, Instituto Agroalimentario de Aragón (IA2), Universidad de Zaragoza-CITA and IIS Aragón, Zaragoza, Spain

Currently, tests of prion infectivity require an in vivo model for their propagation. These expensive and time-consuming experiments are carried out in vertebrate hosts, typically in rodents. This article offers  a sound methodological overview of an alternative Drosophila bioassay developed by the authors. The Drosophila bioassay demonstrates prion-induced toxicity in the level of performance (negative geotaxis) and lifespan when subjected to primary transmission with sheep scrapie material. Bona fide acumulation of infectious prions is demonstrated by secondary transmission to PrP-expressing flies and mice using fly head homogenates from primary infection. Importantly, the described bioassay is sufficiently sensitive to detect sheep with prion disease using plasma from pre-clinical (non-symptomatic) animals. In the future, this could lead to a fast and cost-efficient blood test to diagnose human prion diseases, as well as potentially zoonotic animal diseases. Furthermore, given the ease of transgenesis in Drosophila, the described methodology could possibly be used  to provide insight for potential transmissibility of other protein misfolding disorders such as Alzheimer’s and Parkinson’s diseases. 

 As the aim is to reduce, replace and refine, the main target audience for this methodology would probably be laboratories working with more sentinent mammalian models of prion diseases. In order to facilitate the adoption the invertebrate model for those laboratories not used to work with flies it would be useful to provide an illustrative figure describing the principles of the described bioassay, including the Gal4-system used.  It would also be good mention stock numbers of used fly strains (driver lines and those used for transgenesis) to indicate  that they can be easily obtained from Drosophila stock centres such as BDSC.

**Are a suitable application and appropriate end-users identified?**

 Yes

**If applicable, is the statistical analysis and its interpretation appropriate?**

 Not applicable

**Are the 3Rs implications of the work described accurately?**

 Yes

Is the rationale for developing the new method (or application) clearly explained?

Yes

Is the description of the method technically sound?

Yes

Are the conclusions about the method and its performance adequately supported by the findings presented in the article?

Yes

If any results are presented, are all the source data underlying the results available to ensure full reproducibility?

No source data required

Are sufficient details provided to allow replication of the method development and its use by others?

Yes

Reviewer Expertise:

Neurodegenerative diseases, amyotrophic lateral sclerosis, prion diseases, Drosophila genetics

I confirm that I have read this submission and believe that I have an appropriate level of expertise to confirm that it is of an acceptable scientific standard.
